# Genome Sequence of *Trichoderma lixii* MUT3171, A Promising Strain for Mycoremediation of PAH-Contaminated Sites

**DOI:** 10.3390/microorganisms8091258

**Published:** 2020-08-20

**Authors:** Francesco Venice, Domenico Davolos, Federica Spina, Anna Poli, Valeria Paola Prigione, Giovanna Cristina Varese, Stefano Ghignone

**Affiliations:** 1 Institute for Sustainable Plant Protection (IPSP)–SS Turin—National Research Council (CNR), Viale Mattioli 25, 10125 Turin, Italy; francesco.venice@ipsp.cnr.it (F.V.); stefano.ghignone@ipsp.cnr.it (S.G.); 2 Department of Technological Innovations and Safety of Plants, Products and Anthropic Settlements (DIT), INAIL, Research Area, Via R. Ferruzzi 38/40, 00143 Rome, Italy; d.davolos@inail.it; 3 Department of Life Sciences and System Biology, University of Turin, Viale Mattioli 25, 10125 Turin, Italy; federica.spina@unito.it (F.S.); anna.poli@unito.it (A.P.); valeria.prigione@unito.it (V.P.P.)

**Keywords:** Hypocreaceae, bioremediation, aromatic hydrocarbons, comparative genomics

## Abstract

Mono- and polycyclic aromatic hydrocarbons (PAHs) are widespread and recalcitrant pollutants that threaten both environmental and human health. By exploiting the powerful enzymatic machinery of fungi, mycoremediation in contaminated sites aims at removing a wide range of pollutants in a cost-efficient and environmentally friendly manner. Next-generation sequencing (NGS) techniques are powerful tools for understanding the molecular basis of biotransformation of PAHs by selected fungal strains, allowing genome mining to identify genetic features of biotechnological value. *Trichoderma lixii* MUT3171, isolated from a historically PAH-contaminated soil in Italy, can grow on phenanthrene, as a sole carbon source. Here, we report the draft genome sequence of *T. lixii* MUT3171 obtained with high-throughput sequencing method. The genome of *T. lixii* MUT3171 was compared with other 14 *Trichoderma* genomes, highlighting both shared and unique features that can shed a light on the biotransformation of PAHs. Moreover, the genes potentially involved in the production of important biosurfactants and bioactive molecules have been investigated. The gene repertoire of *T. lixii* MUT3171 indicates a high degrading potential and provides hints on putative survival strategies in a polluted environment.

## 1. Introduction

The fungal genus *Trichoderma* (Ascomycota, Sordariomycetes, Hypocreaceae) gathers successful colonizers of very diverse environments and is found wherever decaying plant material is available. Species from this genus are mostly known as biocontrol agents, capable of protecting plants from pathogens [1] by producing powerful antimicrobial secondary metabolites [2]. *Trichoderma* spp. have also become of biotechnological interest due to their diversified enzymatic arsenal that facilitates their adaptability to a wide range of substrates [3]. These features have been developed throughout an ancient history of environmental opportunism that turned *Trichoderma* spp. into efficient and competitive generalists, through the constant interaction with different organisms [4]. For example, the carbohydrate-active enzymes (CAZymes) repertoire that characterizes *Trichoderma* spp. as efficient phytosaprotrophs was likely acquired ancestrally through mycoparasitism of fungal phytopathogens [5]. These well-developed metabolic pathways enable *Trichoderma* spp. to grow even in highly polluted environments [6], a capacity that is supported by the presence in their genome of genes encoding for multicopper laccases, peroxidases, and ring-cleavage dioxygenases.

Several environments are subjected to a variety of pollution sources, largely due to improper human activities. Mycoremediation in contaminated sites aims at removing hazardous pollutants in a cost-efficient and environmentally friendly manner. Increasing evidence revealed that the success of filamentous fungi in mycoremediation is aided by their ability to make pollutants more bioavailable, through the production of biosurfactants [7,8]. However, only a few studies have revealed the presence of biosurfactant-producing *Trichoderma* strains [9,10]. The production of bioactive compounds by a fungal strain is another important parameter when assessing mycoremediation potential: it redirects nutrient flux from decomposed wastes into molecules that are beneficial to the fungus, guaranteeing competitiveness of the strain and promoting co-metabolization of chemicals, but it also represents a drawback in terms of safety application [11,12].

Here, we investigate the genome of *Trichoderma lixii* MUT3171, isolated from a highly polluted environment. Through the comparison with other sequenced *Trichoderma* and by investigating the peculiar genetic traits of *T. lixii* MUT3171, we bring perspectives on its hypothetical application in mycoremediation, and present a novel genomic resource, as no other *T. lixii* genomic sequences are currently available in public databases.

## 2. Materials and Methods

### 2.1. Microorganism and Growth Conditions

*Trichoderma lixii* MUT3171 was isolated from a site with a long history of industrial exploitation in Fidenza, Italy (N44.866126 W10.074016): the main contaminants were polycyclic aromatic hydrocarbons (PAHs), benzene, toluene, three xylene isomers (BTEX), and alkanes (Table S1), with a total hydrocarbon content of 378 mg/kg of soil.

The strain is deposited at Mycotheca Universitatis Taurinensis (MUT, www.mut.unito.it) of the Department of Life Sciences and Systems Biology, University of Torino, Torino (Italy). The soil dilution plate method was used to isolate microorganisms on a selective medium (soil suspensions were plated on minimal Czapek medium supplemented with hydrocarbons as sole carbon source). *Trichoderma lixii* MUT3171 was isolated in the presence of phenanthrene at 200 mg/L. Mycelium for DNA extraction was grown on 2% malt extract agar at 25 °C for 7 days.

The identification at species level was carried out through morphological and molecular analyses. The internal transcribed spacer region (*ITS*) and translation elongation factor 1-alpha (*TEF1-alpha*) gene sequences obtained for *T. lixii* MUT3171 are available at GenBank NCBI under the accession numbers MF305834 and MT435114, respectively.

### 2.2. Laccases and Biosurfactants Analysis

The ability of *T. lixii* MUT3171 to produce extracellular laccases was evaluated using a color-based method. *Trichoderma lixii* MUT3171 was inoculated in 2% malt extract agar supplemented with 2,2′-azino-bis (3-ethylbenzothiazoline-6-sulphonic acid) (ABTS) (1 mM). In the presence of laccase activity, the colorless medium turns dark green. Plates were incubated at 24 °C for 7 days.

For biosurfactants production, a qualitative drop collapse test and oil dispersion assay were performed as described by Boudour and Miller-Maier [13], and by Morikawa et al. [14]. *Trichoderma lixii* MUT3171 was inoculated in a modified mineral salt medium: soybean oil (4% *w/w*) was added to stimulate the surfactants producing metabolism. The flasks were incubated at 24 °C and 120 rpm. The culture broth was separated from the mycelium by centrifugation (10 min, 4 °C and 5000 rpm) and aliquots of the supernatant were analyzed. Positive controls were set up with Tween 80, whereas negative controls were performed using deionized water.

### 2.3. DNA Extraction, Sequencing and Bioinformatics Analyses

Total DNA was extracted from mycelial samples of *T. lixii* MUT3171 using the QIAamp DNA Microbiome Kit (Qiagen, Germany). DNA quality and concentration were assessed with Nanodrop 2000 (Thermo Fisher Scientific, Wilmington, DE, USA), and 1 ng DNA was used for library preparation. The paired-end genomic libraries (2 × 250 bp) were built using the MiSeq v. 3 reagents (600 cycles) and sequenced at EUROFINS GENOMA Group (Rome, Italy).

Reads quality was assessed with fastQC v.0.11.9 [15], and adapters removal was performed with Trimmomatic v.0.38 [16] allowing up to 3 mismatches and setting 20 and 8 as palindrome and simple clip threshold, respectively. The surviving reads (~99.2%) were de novo assembled using SPAdes v. 3.11 [17] with default parameters, in combination with BayesHammer (distributed with SPAdes latest version) [18] for reads error correction. The mitochondrial genome was also reconstructed to isolate the nuclear component. MITObim v. 1.9.1 [19] was used for the reconstruction of the mitochondrial sequence, which was then annotated with MFannot [20] and GeSeq [21]. GeSeq was run using the mitochondria of *Trichoderma asperellum* (NC_037075.1), *Trichoderma gamsii* (NC_030218.1), *Trichoderma hamatum* (NC_036144.1) and *Trichoderma reesei* (NC_003388.1) as reference sequences.

Nuclear genome statistics were calculated using QUAST v. 4.5 [22]. RepeatModeler v. 2.0.1 [23] and RepeatMasker v. 4.1.0 [24], both using NCBI/RMBLAST v. 2.10.0+ as a search engine, were used to identify repetitive and low-complexity regions within the assembly. Ribosomal RNA (*rRNA*) and transfer-RNA (*tRNA*) were predicted with RNAmmer v.1.2 [25] and tRNAscan-SE v. 2.0.5 [26], respectively.

De novo gene prediction was performed with Augustus v. 2.5.5 [27] using *Fusarium graminearum* as training species. Proteome completeness was calculated with BUSCO v. 3.0.1 [28] using the Sordariomycetes conserved gene set. Secretome prediction was performed following the pipeline described in Pellegrin et al. [29]. Briefly, proteins carrying a secretion signal were identified with SignalP v. 5.0 [30] and TargetP [31], while proteins with a transmembrane domain or with a signal for endoplasmic reticulum permanent retention (PS00014) were identified and discarded with TMHMM v. 2.0 [32] and by ScanProsite [33], respectively. Subsequently, Wolf PSORT v. 0.2 was used to discard proteins directed to organelles [34]. CAZymes were identified with HMMER v. 3.3 [35] and the dbCAN Database v. 7 [36]. For the comparative genome analysis, we performed orthology inference through basic local alignment search tool (BLAST) searches, MCL clustering, multiple sequence alignments and genome-wide phylogenies with OrthoFinder v. 2.3.7 [37], targeting the proteomes of 14 currently available genomes from the genus *Trichoderma* (including *T. lixii* MUT3171; accessions shown in Table S2). We used the “-m msa” OrthoFinder option that computes multiple sequence alignments and reconciles gene trees for each group of orthologs, combining MAFFT v. 6.240 [38] and FastTree v. 2.1 [39], respectively. For each proteome, an InterProScan v. 5.38.76 [40] full analysis was performed. The function of each protein was inferred by integrating the automatic annotation with a manual curation step: the annotations were considered credible only if multiple proteins in the same group of orthologs shared at least one characterizing domain. *Trichoderma lixii* MUT3171 proteins that lacked orthology in relation to those of the other *Trichoderma* species examined in the present study were analyzed with BLASTp against the NCBI nr and conserved domain database (CDD) [41] and visualized, together with their best BLAST hits, with Geneious v. 2020.1.2 (https://www.geneious.com/download).

Secondary metabolites gene clusters identification and characterization have been performed with antiSMASH v. 5.1.1 [42] and BIG-SCAPE v. 1.0 [43].

## 3. Results and Discussion

### 3.1. Trichoderma lixii MUT3171 Genomic Parameters

The results of colony morphology and phylogenetic analyses based on *ITS* and *TEF1-alpha* indicated that *Trichoderma lixii* MUT3171 is closely related to *Trichoderma lixii* (based on BLASTn results with MF305834 and MT435114 as query).

The final assembly for *T. lixii* MUT3171 nuclear genome is approximately 41 Mbp and possesses 11,923 protein-coding genes (Table 1). The circular mitochondrial genome of *T. lixii* MUT 3171 (GenBank accession: MT495248) is 29.79 kbp in length with GC% 27.42. The mitochondrial assembly contains the gene for the ribosomal protein S3 (rps3), 14 core genes that encode proteins involved in oxidative phosphorylation and electron transport, and a highly degraded copy of the 3′ fragment of the NAD4 gene. Moreover, mitochondrial genes include the small and the large subunit of *rRNA*, as well as 25 *tRNA* genes. The gene order is conserved among other *Trichoderma* mitochondrial sequences (NC_037075.1, NC_030218.1, NC_036144.1, NC_003388.1).

The nuclear genome contains single copies of 18S and 28S *rRNAs*, 44 8S *rRNAs* (of which 16 organized in clusters), 182 *tRNAs*, and five pseudogenes. Around 28% of the nuclear genome is repeated. The nuclear genome encodes for 472 secreted proteins, of which around 40% are smaller than 300 amino acids, making them candidate secreted effectors. More details on nuclear genome characteristics are provided in Table 1.

### 3.2. Orthology-Based Survey of T. lixii MUT3171 Degradative Enzymes

**Figure 1:**
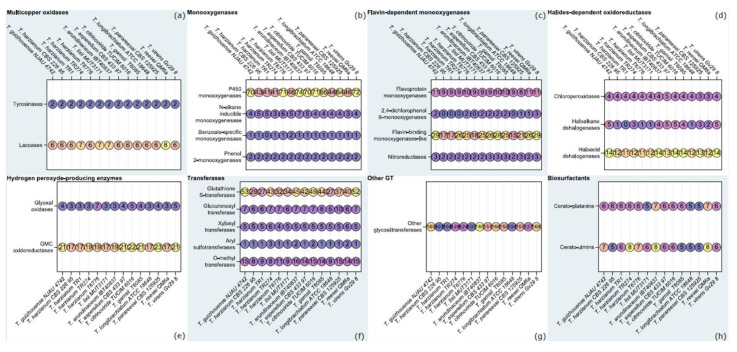
Degradative potential of *Trichoderma* species, compared with *Trichoderma lixii* MUT3171: Comparative genome analysis of *T. lixii* MUT3171, and 14 sequenced *Trichoderma* species (more details are provided in Table S2) in order to investigate well-supported enzymatic categories involved in the degradation of pollutants [44]. In each box and for each species, the number of orthologs of a specific enzyme class is reported.

We have investigated the degradative potential of *T. lixii* MUT3171 by characterizing the enzyme-encoding genes that may participate in the cleavage of PAHs and other pollutants found in the sampling site. The genome of *T. lixii* MUT3171 was compared with those of other *Trichoderma* strains deposited in NCBI (to January 2020). We established an orthology relationship between the gene products of 14 species from the genus (including *T. lixii* MUT3171), i.e., we identified oxidoreductases, CAZymes, and biosurfactants, that probably derive from a common ancestor within the species analyzed in this study [37]. Figure 1 reports the sum of the proteins in each group of orthologs containing the enzymes involved in the cleavage of PAHs and other pollutants [44]. Our analysis confirmed that among the isolates belonging to the *Trichoderma harzianum* complex (*Trichoderma guizhouense* NJAU 4742, *T. lixii* MUT3171, and four *T. harzianum* strains), numbers of putative detoxifying enzymes are highly comparable, whereas at the genus level they are not. Multicopper oxidases (Figure 1a) seem to be conserved in their number at genus level, but *T. lixii* MUT3171 and *T. harzianum* TR274 have 7 laccases, while other isolates from the *T. harzianum* complex have 6. Both *T. lixii* MUT3171 tyrosinases are extracellular, while most of laccases seem to be cytoplasmic (6 out of 7). Biochemical evidence of intracellular laccases in *Trichoderma* is completely missing. Only thanks to molecular phylogenetic analyses, their occurrence in *T. harzianum* and *T. reesei* genome have been demonstrated [45]. Extracellular laccases are mostly known from *Trichoderma atroviride, T. harzianum* and *T. asperellum* [46,47,48]. Recent studies have suggested that *Trichoderma* strains are barely capable of producing extracellular laccases constitutively. As found for *Trichoderma camerunense* [9] and *Trichoderma koningiopsis* [49], *T. lixii* MUT3171 showed a minimal laccase activity: the oxidation of ABTS in solid plate test was marginally appreciable (halo diameter approx. 2 cm). However, laccase gene transcription is highly sensitive to xenobiotics-related stress. The production of laccases in *Trichoderma* can be stimulated by the presence of aromatic compounds as alachlor [50], guaiacol [51], and pyrene [52], as a direct response of the fungus to their toxicity. Laccases may be involved in the primary attack of PAHs biodegradation, catalyzing the oxygenation and the oxidative cleavage of aromatic structure [52].

The entire degradation pathway may involve other enzymes such as dioxygenases [52,53] and cytochrome P450 complex [50]. Remarkably, monooxygenases were the most abundant enzymatic class in the *T. lixii* MUT3171 genome (Figure 1b). Our analysis demonstrated that P450 monooxygenases are the largest subclass. Although they are involved in a variety of processes, including secondary biosynthesis of metabolites, some of them contain domains indicating their specificity to alkanes and benzoate. In particular, *T. lixii* MUT3171 has two benzoate-specific monooxygenases, while other *Trichoderma* have one or none. As for the other *Trichoderma* analyzed in this study, flavin-dependent monooxygenases of *T. lixii* MUT3171 are the second most abundant class (Figure 1c)*,* being mostly composed by flavin-binding monooxygenases-like. No significant increase in gene numbers involved in halides-dependent peroxidation or dehalogenation was observed (Figure 1d). In this enzymatic class, the four chloroperoxidases of *T. lixii* MUT3171 are predicted to be extracellular. As peroxidases activity requires the production of H_2_O_2_, we also searched for enzymes involved in this process, i.e., glyoxal oxidases and Glucose-Methanol-Choline (GMC) oxidoreductases [44], including glucose oxidases, cellobiose dehydrogenases, and aryl alcohol dehydrogenases (Figure 1e). Even in this case, *T. lixii* MUT3171 genome is comparable to that of the other *Trichoderma* species examined in this study.

Additionally, degradation of PAHs strongly relies on phase-II enzymes that mainly consist of transferases initiating the catabolism of PAHs byproducts [44]. Our results confirmed that the analyzed *Trichoderma* genomes possess a large number of glutathione S-transferases and glycosyltransferases (Figure 1f). The expansion of the glycosyltransferase family has a key role in the success of *Trichoderma* as degraders of plant material [4], and our analysis revealed that this feature is unaltered in *T. lixii* MUT3171 (Figure 1f,g). Glycosyltransferases may participate to phase II reactions in the PAHs degradation pathway [44]: after the initial attack by oxidoreductases, they transfer an activated sugar residue to the hydroxyl groups of the PAHs metabolites. Conjugates are formed, such as O-glucosides, O-glucuronides, and O-xylosides, that are not normally degraded further and may be secreted.

The knowledge of biosurfactants production by fungi is still very scarce, although these compounds represent a key factor in the PAHs transformation by enhancing the PAHs bioavailability into the soil [54]. Only a few studies focused on these metabolites within the *Trichoderma* genus. To date, biosurfactant and bioemulsifier activities were observed in *T. camerunense* [54] and *T. reesei* [55]. A class II hydrophobin produced by *T. reesei* is the only characterized biosurfactant [55]. Likewise, *T. lixii* MUT3171 has the genetic potential to produce biosurfactants, specifically the fungal hydrophobins cerato-platanins and cerato-ulmins (Figure 1h), whose role in mycoremediation is now emerging [7,56]. In agreement with this finding, *T. lixii* MUT3171 produced extracellular biosurfactants in in vitro tests. Qualitative drop collapse test and oil dispersion assay gave positive results: the drop was partially flattering and an unequivocal clarification halo (2.5 cm diameter) was observed.

### 3.3. Unique Genetic Features of T. lixii MUT3171

**Figure 2:**
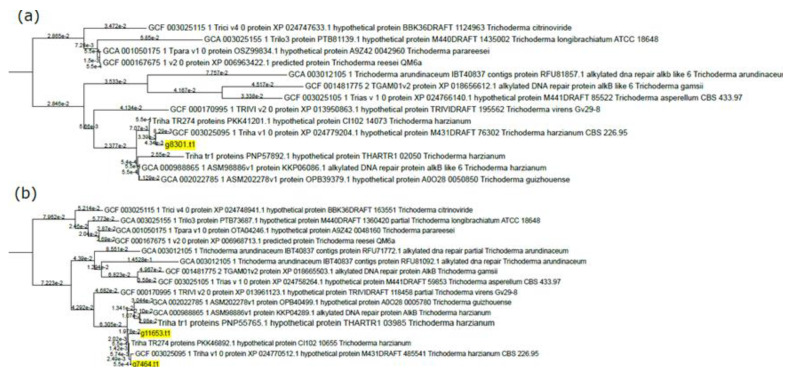
DNA alkylation damage-related genes in *T. lixii* MUT3171 and their relationship with other *Trichoderma* species: Distance tree of orthologous *Trichoderma* enzymes with a putative function in DNA protection against alkylation damages; distances are provided in scientific notation. The distance trees were generated through the OrthoFinder 2.0 pipeline. *Trichoderma lixii* MUT3171 sequences are highlighted in yellow. (**a**) g8301.t1 showed a very low level of divergence compared with its closest ortholog found in *T. harzianum* TR1, (**b**) g11653.t1 revealed a higher level of divergence compared with putative orthologs and the paralog g7464.t1.

Since genome mining revealed a rough overlap between *T. lixii* MUT3171 and other sequenced *Trichoderma* species in terms of degrading enzyme composition, we focused on other genetic traits that may guarantee the survival of *T. lixii* MUT3171 in petroleum-polluted environments. Three gene sequences (g11653.t1, g7464.t1, and g8301.t1) from *T. lixii* MUT3171 possess a PF13532 domain that indicates their involvement in the repair of DNA following alkylation damages. These enzymes, such as AlkB, mostly received attention in bacteria inhabiting petroleum-contaminated soils [57]. From a functional point of view, they are dioxygenases that require ketoglutarate and iron (2Fe-OG dioxygenases). The orthology-based phylogenetic reconstruction indicates that the products of these three genes are gathered in two groups of orthologs (Figure 2). While g8301.t1 (Figure 2a) might have limited divergence from its closest *T. harzianum* homolog (GCF03025095.1), the phylogenetic reconstruction highlights a stronger differentiation between g11653.t1 and g7464.t1 (Figure 2b), gathered in the same group of orthologs. In this group, only *T. lixii* MUT3171 has a double copy of this gene, which may suggest a duplication event that led to functional diversification. The presence of these enzymes may be linked to the survival of *T. lixii* MUT3171 in the polluted site that is also contaminated with n-alkanes.

**Figure 3:**
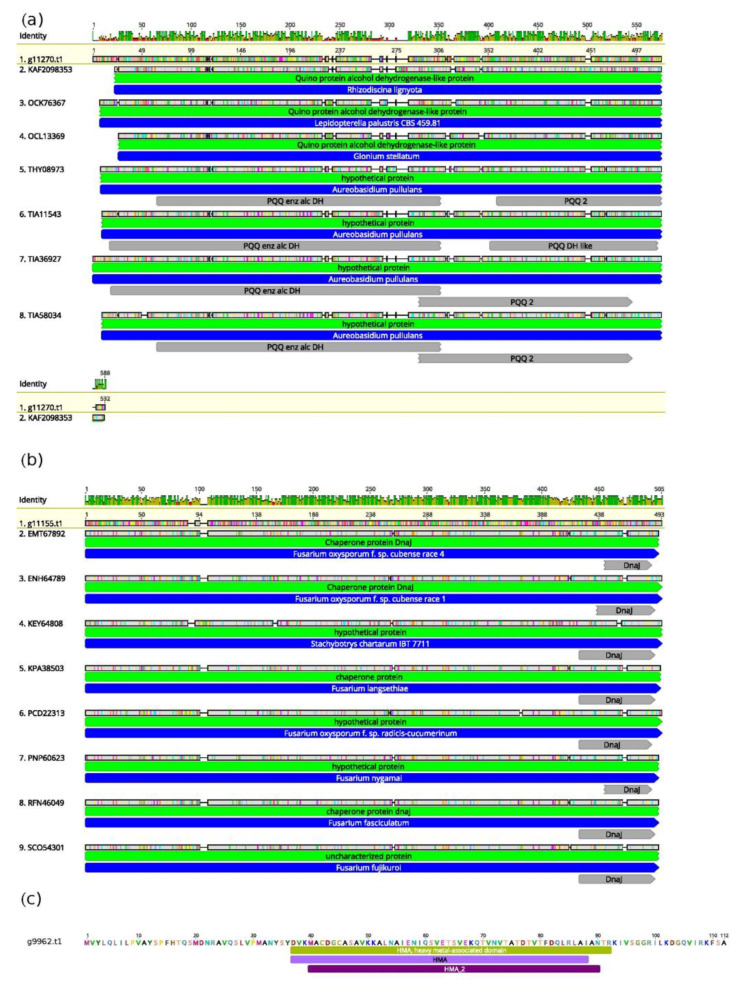
*Trichoderma lixii* MUT3171 peculiar genomic features linked to survival in the extreme environment: Graphical representation of the basic local alignment search tool (BLAST) and conserved domain database (CDD) search results obtained for *T. lixii* MUT3171 proteins lacking homology compared with those from *Trichoderma* protein sequences available at GenBank, including (**a**) a quinoprotein alcohol dehydrogenase found in Dothideomycetes, (**b**) a DnaJ chaperone with homology in *Fusarium* species, and (**c**) a protein associated with heavy metal tolerance but without homology with any sequence deposited at the GenBank database. For each query sequence (highlighted in yellow), green bars are top BLAST hits, upper panels are the degree of amino acid conservation throughout all the sequence. Blue bars indicate the taxonomic affiliation of each sequence, the interrupted bars above each target sequence indicate its BLAST alignment against the *T. lixii* MUT3171 query. In these bars, the gray color represents amino acid identity, and colored points indicate amino acid substitutions. The functional domains identified by CDD search are provided below bars and are indicated in gray or violet colors.

Notably, the genome of *T. lixii* MUT3171 also encodes for several proteins that lack homology and cannot be compared with those found in other species of *Trichoderma* (Figure 3). For example, we found a quinoprotein alcohol dehydrogenase (Figure 3a) with a strong homology with the one of *Rhizodiscina lygniota* (Dothideomycetes), a poorly studied wood saprotroph [58]. Indeed, the top BLAST hits for this *T. lixii* MUT3171 protein include only Dothideomycetes, with *Lepidopterella palustris*, and *Glonium stellatum*, both wood decayers [59], together with the yeast *Aureobasidium pullulans*. However, little is known about the degrading potential of fungal quinoprotein alcohol dehydrogenases. Few reports, largely based on bacteria, have provided evidence of a specific role in phenanthrene degradation [60].

Another example is the chaperone protein of the DnaJ class in *T. lixii* MUT3171 (Figure 2b) that has several homologs in *Fusarium oxysporum,* but not in *Trichoderma.* The role of DnaJ proteins consists mainly in the maintenance of endoplasmic reticulum stability under unfolded protein response, enhancing virulence in *F. oxysporum* and *Ustilago maydis* [61]. These proteins may also be involved in the tolerance against toxic compounds, as demonstrated in the black yeast *Cladophialophora immunda* that strongly activated a DnaJ-mediated response to toluene [62]. *Trichoderma lixii* MUT3171 genome also encodes for a protein (g9962.t1, Figure 3c) with no known homologs in public databases (as of January 2020). This protein contains a domain associated with the tolerance to heavy metals by fungi, plants, and bacteria [63,64] and it is found in both chaperones and transporters.

### 3.4. Prediction of Secondary Metabolism Genes in T. lixii MUT3171

**Figure 4:**
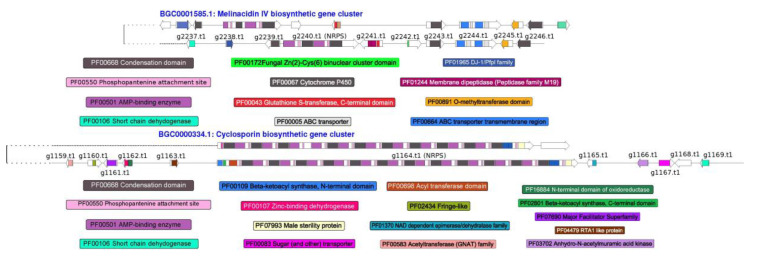
Non-ribosomal peptide synthases (*NRPS*) genes in *T. lixii* MUT3171: Graphical representation of two *NRPS* putative gene clusters found in *T. lixii* MUT3171 showing similarities with melinacidin IV and cyclosporin biosynthetic gene clusters, as provided by the MIBiG repository. For each gene cluster, the genes and their functional domains are reported. Only domains found in *T. lixii* MUT3171 genes are reported.

We identified candidate genes for the production of secondary metabolites and bioactive molecules in *T. lixii* MUT3171 genome by means of the antiSMASH pipeline. Numerous recent studies have revealed that the biosynthetic genes are often organized in transcriptionally co-regulated clusters [65], and the biosynthetic process is driven by non-ribosomal peptide synthases (*NRPS*), polyketide synthases (*PKS*) and their hybrids (*hybrid NRPS-PKS*). In *T. lixii* MUT3171, we identified 23 *PKS*, 19 *NRPS*, and 8 *NRPS-PKS hybrids*, often co-localized on the genome with P450 monooxygenases, FAD-binding proteins, general substrates transporters, and multicopper oxidases. Nine siderophores were predicted as well, indicating that *T. lixii* MUT3171 has a relevant potential in iron sequestration, a process associated with microbial competition, resistance to oxidative stress, and development under limiting conditions [66]. Most of the gene clusters from all classes in *T. lixii* MUT3171 cannot be further annotated as their composition is not similar to any known biosynthetic gene cluster. Nevertheless, we were able to infer a distance-based classification of two *NRPS*, three *PKS*, and one *NRPS-PKS*. Through multiple sequence alignments, the BIG-SCAPE analysis calculated the distances between the analyzed sequences and those present in the MIBiG repository [67] of known biosynthetic genes clusters. This repository contains the minimal composition of each gene cluster, while antiSMASH also includes flanking genes, which results in larger clusters and may decrease the final similarity score. Two *NRPS* genes of *T. lixii* MUT3171 (Figure 4) had at least 45% identity with the antibiotic melanicidin IV and the immunosuppressant cyclosporin biosynthetic clusters from *Escovopsis weberi* and *Tolypocladium inflatum* NRRL8044, respectively (BGC0001585 and BGC0000334 in the MIBiG repository). While the production of cyclosporin A is known in *T. harzianum* [68], melinacidin IV has never been reported in *Trichoderma* species, although phylogenetic results and lifestyle relationships between *Escovopsis* and *Trichoderma* [69] may provide insights into this putative trait.

**Figure 5:**
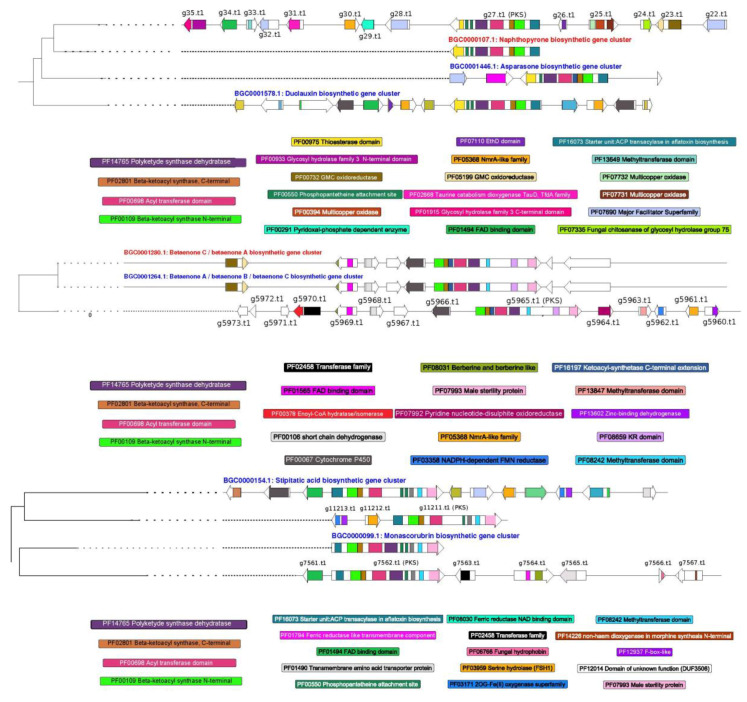
Polyketide synthases (*PKS*) genes in *T. lixii* MUT3171: Graphical representation of three *PKS* gene clusters found in *T. lixii* MUT3171 showing similarities with naphthopyrone, betaenones, stipitatic acid, and monoascorubrin biosynthetic gene clusters provided by the MIBig repository. For each gene cluster, the genes and their functional domains are reported. Only domains found in *T. lixii* MUT3171 genes were reported.

Among the *PKS* clusters (Figure 5), three gene clusters of *T. lixii* MUT3171 resemble those for the production of naphthopyrone in *Aspergillus nidulans* FGSC A4 (BGC0000107), betaenone in *Phoma betae* (BGC0001264 and BGC0001280), monoascorubrin in *Talaromyces marneffei* (BGC0000099) and stipitatic acid in *Talaromyces stipitatus* ATCC 10500 (BGC0000154). Naphthopyrones are pigments that protect Ascomycetes from a wide range of predators [70], but their derivatives can display cytotoxic activity [71]. Betaenones are phytotoxic and their production was not confirmed in *Trichoderma*. The candidate biosynthetic gene cluster for the production of the antimalarial drug stipitatic acid appears incomplete in *T. lixii* MUT3171, missing an essential cytochrome P450 and a FAD-binding protein [72]. The genomic region potentially involved in the biosynthesis of monoascorubrin, a red pigment historically used as food colorant [73], contains more genes than the reference BGC0000099, but the central *PKS* genes (g7562.t1 in *T. lixii* MUT3171) are nearly identical.

**Figure 6:**
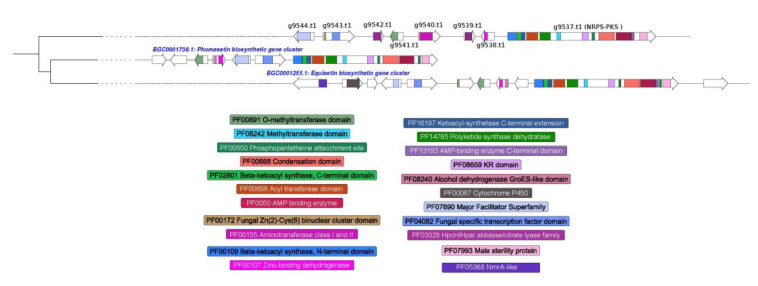
*Hybrid NRPS-PKS* genes in *T. lixii* MUT3171: Graphical representation of a *hybrid NRPS-PKS* gene cluster found in *T. lixii* MUT3171 showing similarities with equisetin and phomasetin biosynthetic gene clusters, as provided by the MIBig repository. For each gene cluster, the genes and their functional domains are reported. Only domains found in *T. lixii* MUT3171 genes were reported.

The detection of a *hybrid NRPS-PKS* (Figure 6) in *T. lixii* MUT3171 indicates setins production. These data are in accordance with other studies that demonstrated their production in *Trichoderma* [74]. Setins include phomasetin and equisetin that are produced by well-known gene clusters found in *Pyrenochaetopsis* sp. and *Fusarium heterosporum*, respectively. The gene clusters of *T. lixii* MUT3171 share with them an O-methyltransferase (g9541.t1), a major facilitator transporter (g9544.t1) and a zinc-binding dehydrogenase (g9538.t1). Evidence that phomasetin and equisetin may be antibiotics with inhibitory activity against HIV-1 integrase has been provided [75].

## 4. Conclusions

In this work, the genome mining of *T. lixii* MUT3171 highlighted peculiar genetic traits that could explain its ability to inhabit extremely polluted environments and to grow on phenanthrene as the sole carbon source. *Trichoderma lixii* MUT3171 shares a similar degrading potential with other *Trichoderma* species, being capable of producing a large number of multicopper oxidases, monooxygenases, and biosurfactants. The genetic features of *T. lixii* MUT3171 and the comparison to the aforementioned *Trichoderma* species lead to some conclusions that may be drawn. It is possible to speculate that the *T. lixii* MUT3171 genome has not undergone simplification or loss of functions because of the extreme lifestyle in a polluted environment, at least in term of gene number, secreted proteins repertoire and content of CAZymes as glycosyltransferases, that are iconic in the *Trichoderma* genus [4]. Moreover, the ability of *T. lixii* MUT3171 to survive in a highly polluted environment may not only depend on its degradative enzymes. Indeed, genome mining revealed several traits that are unique to *T. lixii* MUT317, such as the presence of specialized mechanisms for DNA repair, the protection from protein unfolding and even the tolerance to heavy metals. Given the lack of homology with other *Trichoderma* sequences, these genes might be the result of horizontal gene transfer, a widespread phenomenon in the *Trichoderma* genus [5]. *Trichoderma lixii* MUT3171 encodes for 58 secondary metabolites gene clusters that probably guarantee its competitiveness and survival. These genetic features are studied not only because they can provide adaptation to an extremophilic lifestyle, but also due to their biotechnological potential and economical value. Our results indicated the involvement of iron in the fungal tolerance to pollutants, as it is crucial for the functioning of DNA repair enzymes (2Fe-OG dioxygenases) and antioxidant enzymes that contain iron-sulfur clusters. This observation is also confirmed by the presence in *T. lixii* MUT3171 genome of 9 genes encoding for siderophores. This finding opens the possibility of using direct iron supplementation, or bioaugmentation using mixed microbial consortia to increase iron availability and enhance the fungal performance [76].

These observations emphasize the perspective of testing degradative ability of *T. lixii* MUT3171, making it a good candidate for the treatment of PAH-contaminated soils.

## Figures and Tables

**Table 1:** Nuclear genome statistics: Parameters and content of the nuclear genome assembly of *Trichoderma lixii* MUT3171.

Parameter	Estimated Value			Total Genome Coverage
Assembly size	~40.89 Mbp			-
Number of scaffolds	2142			-
G+C content	~49.4%			-
Genome gaps (Ns)	~0.005% (2045 bp)			-
N50/L50	77,948 kbp/156 scaffolds			-
Number of genes	11,923			-
tRNAs	182			-
rRNAs	**8 s**	**18 s**	**28 s**	-
	44	1	1	-
Small RNAs	14			-
Secreted proteins	472			-
Satellites	14			0.02%
Simple repeats	8820			0.89%
Low complexity regions	1528			0.19%
Unclassified transposon sequences	1499			0.71%
Retrotransposon sequences	**SINEs**	**LINEs**	**LTR (Gypsy/DIRS1)**	0.83%
	15	226	536	
DNA transposon sequences	**hobo-Activator**	**Tc1-IS630-Pogo**	**Others**	0.18%
	44	49	167	

## Data Availability

The WGS libraries, the mitochondrial and nuclear assemblies of *T. lixii* MUT3171 are available at GenBank (NCBI) under the BioProject PRJNA514353, and BioSample ID SAMN10723660.

## Supplementary Materials

The following are available online at https://www.mdpi.com/2076-2607/8/9/1258/s1, **Table S1.** Pollutants concentration: list of the compounds detected in the contaminated soil, in which *Trichoderma lixii* MUT3171 was isolated. The concentration is expressed in mg/kg of dry soil. TPH: total petroleum hydrocarbons, **Table S2**. Strains used for comparison: list of *Trichoderma* species used in the comparative analysis, with relative GenBank accessions and references.
