# Supplementary material for: Genome Sequence of Trichoderma lixii MUT3171, A Promising Strain for Mycoremediation of PAH-Contaminated Sites
Source: Microorganisms. 2020 Aug 20;8(9):1258. doi: 10.3390/microorganisms8091258 (PMC7570066; doi:10.3390/microorganisms8091258)
Supplement: Supplementary file 1 [file microorganisms-08-01258-s001.pdf]

**Table S1. Pollutant concentration**: List of the compounds detected in the contaminated soil, in which *Trichoderma lixii* MUT3171 was isolated. The concentration is expressed in mg/kg of dry soil. TPH: total petroleum hydrocarbons.

| Pollutant | Concentration |
|---|---|
| 2-metilnaphthalene | 50.00 |
| 1-metilnaphthalene | 28.26 |
| naphthalene | 62.81 |
| acenaphtilene | 0.44 |
| acenaphthene | 10.96 |
| fluorene | 11.83 |
| phenanthrene | 96.28 |
| anthracene | 6.67 |
| fluoranthene | 51.01 |
| pyrene | 32.91 |
| benzo(a)anthracene | 10.11 |
| chrysene | 5.97 |
| benzo(b+j)fluoranthene | 3.96 |
| benzo(k)fluoranthene | 1.50 |
| benzo(a)pyrene | 1.93 |
| indeno[1,2,3-cd]pyrene | 1.24 |
| dibenzo(ac+ah)anthracene | 0.32 |
| benzo(ghi)perylene | 1.02 |
| dibenzo(al)pyrene | 0.50 |
| dibenzo(ae)pyrene | 0.14 |
| dibenzo(ai)pyrene | 0.05 |
| dibenzo(ah)pyrene | <0.01 |
| TPHs | 377.91 |

**Table S2. Strains used for comparison**: List of *Trichoderma* species used in the comparative analysis, with relative GenBank accessions and references.

| Species | Accession | References |
|---|---|---|
| *Trichoderma guizhouense* NJAU 4742 | GCA_002022785.1 | [1] |
| *Trichoderma harzianum* CBS 226 95 | GCA_003025095.1 | [2] |
| *Trichoderma harzianum* TR274 | GCA_002838845.1 | [3] |
| *Trichoderma harzianum* T6776 | GCA_000988865.1 | [4] |
| *Trichoderma arundinaceum* IBT40837 | GCA_003012105.1 | [5] |
| *Trichoderma asperellum* CBS 433 97 | GCA_003025105.1 | [2] |
| *Trichoderma citrinoviride* TUCIM 6016 | GCA_003025115.1 | [2] |
| *Trichoderma gamsii* T6085 | GCA_001481775.2 | [2] |
| *Trichoderma longibrachiatum* ATCC 18648 | GCA_003025155.1 | [2] |
| *Trichoderma parareesei* CBS 125925 | GCA_001050175.1 | [6] |
| *Trichoderma reesei* QM6a | GCA_000167675.2 | [7] |
| *Trichoderma virens* Gv29 8 | GCA_000170995.2 | [8] |
| *Trichoderma harzianum* TR1 | GCA_002894145.1 | [9] |

## References


1. Meng, X.; Miao, Y.; Liu, Q.; Ma, L.; Guo, K.; Liu, D.; Ran, W.; Shen, Q. TgSWO from *Trichoderma guizhouense* NJAU4742 promotes growth in cucumber plants by modifying the root morphology and the cell wall architecture. *Microb. Cell Factories* **2019**, *18*, 148–15, doi:10.1186/s12934-019-1196-8.
2. Druzhinina, I.S.; Chenthamara, K.; Zhang, J.; Atanasova, L.; Yang, D.; Miao, Y.; Rahimi, M.J.; Grujic, M.; Cai, F.; Pourmehdi, S.; et al. Massive lateral transfer of genes encoding plant cell wall-degrading enzymes to the mycoparasitic fungus *Trichoderma* from its plant-associated hosts. *PLoS Genet.* **2018**, *14*, e1007322, doi:10.1371/journal.pgen.1007322.
3. Steindorff, A.S.; Ramada, M.H.S.; Coelho, A.S.; Miller, R.N.G.; Pappasjr, G.J.; Ulhoa, C.J.; Noronha, E.F. Identification of mycoparasitism-related genes against the phytopathogen *Sclerotinia sclerotiorum* through transcriptome and expression profile analysis in *Trichoderma harzianum*. *BMC Genom.* **2014**, *15*, 204, doi:10.1186/1471-2164-15-204.
4. Baroncelli, R.; Zapparata, A.; Piaggeschi, G.; Sarrocco, S.; Vannacci, G. Draft whole-genome sequence of *Trichoderma gamsii* T6085, a promising biocontrol agent of *Fusarium* head blight on wheat. *Genome Announc.* **2016**, *4*, 4, doi:10.1128/genomea.01747-15.
5. Proctor, R.H.; McCormick, S.P.; Kim, H.-S.; Cardoza, R.E.; Stanley, A.M.; Lindo, L.; Kelly, A.; Brown, D.W.; Lee, T.; Vaughan, M.M.; et al. Evolution of structural diversity of trichothecenes, a family of toxins produced by plant pathogenic and entomopathogenic fungi. *PLOS Pathog.* **2018**, *14*, e1006946, doi:10.1371/journal.ppat.1006946.
6. Baroncelli, R.; Piaggeschi, G.; Fiorini, L.; Bertolini, E.; Zapparata, A.; Pè, M.E.; Sarrocco, S.; Vannacci, G. Draft whole-genome sequence of the biocontrol agent *Trichoderma harzianum* T6776. *Genome Announc.* **2015**, *3*, 3, doi:10.1128/genomea.00647-15.
7. Yang, D.; Pomraning, K.R.; Kopchinskiy, A.; Aghcheh, R.K.; Atanasova, L.; Chenthamara, K.; Baker, S.E.; Zhang, R.; Shen, Q.; Freitag, M.; et al. Genome sequence and annotation of *Trichoderma parareesei*, the ancestor of the cellulase producer *Trichoderma reesei*. *Genome Announc.* **2015**, *3*, doi:10.1128/genomea.00885-15.
8. Martinez, D.; Berka, R.M.; Henrissat, B.; Saloheimo, M.; Arvas, M.; Baker, S.E.; Chapman, J.; Chertkov, O.; Coutinho, P.M.; Cullen, D.; et al. Genome sequencing and analysis of the biomass-degrading fungus *Trichoderma reesei* (syn. *Hypocrea jecorina*). *Nat. Biotechnol.* **2008**, *26*, 553–560, doi:10.1038/nbt1403.
9. Kubicek, C.P.; Herrera-Estrella, A.; Seidl, V.; A Martinez, D.; Druzhinina, I.S.; Thon, M.R.; Zeilinger, S.; Casas-Flores, S.; Horwitz, B.A.; Mukherjee, P.K.; et al. Comparative genome sequence analysis underscores mycoparasitism as the ancestral life style of *Trichoderma*. *Genome Boil.* **2011**, *12*, R40, doi:10.1186/gb-2011-12-4-r40.
10. Gardiner, D.; Vos, C.; Kazan, K.; Harvey, P. Commonwealth Scientific and Industrial Research Organisation, Queensland, Australia. Unpublished Work. **2018**.
